# Genetic Diversity in Gorkhas: an Autosomal STR Study

**DOI:** 10.1038/srep32494

**Published:** 2016-09-01

**Authors:** Kiran Preet, Seema Malhotra, Pankaj Shrivastava, Toshi Jain, Shweta Rawat, L. Robert Varte, Sayar Singh, Inderjeet Singh, Soma Sarkar

**Affiliations:** 1 Defence Institute of Physiology and Allied Sciences, Defence Research and Development Organization, Ministry of Defence, Government of India, Lucknow Road, Delhi, 110054, India; 2 State Forensic Science Laboratory, Civil Lines, Sagar, Madhya Pradesh, 470001, India

## Abstract

Genotyping of highly polymorphic autosomal short tandem repeat (STR) markers is a potent tool for elucidating genetic diversity. In the present study, fifteen autosomal STR markers were analyzed in unrelated healthy male Gorkha individuals (n = 98) serving in the Indian Army by using AmpFlSTR Identifiler Plus PCR Amplification Kit. In total, 138 alleles were observed with corresponding allele frequencies ranging from 0.005 to 0.469. The studied loci were in Hardy-Weinberg Equilibrium (HWE). Heterozygosity ranged from 0.602 to 0.867. The most polymorphic locus was Fibrinogen Alpha (FGA) chain which was also the most discriminating locus as expected. Neighbor Joining (NJ) tree and principal component analysis (PCA) plot clustered the Gorkhas with those of Nepal and other Tibeto-Burman population while lowlander Indian population formed separate cluster substantiating the closeness of the Gorkhas with the Tibeto-Burman linguistic phyla. Furthermore, the dataset of STR markers obtained in the study presents a valuable information source of STR DNA profiles from personnel for usage in disaster victim identification in military exigencies and adds to the Indian database of military soldiers and military hospital repository.

The *Gorkha* (also spelt as *Gurkha*) soldiers are a dominant force in the Indian Army who have completed 200 years in the Armed Forces since their integration on April 24, 1815. They are courageous in battle and have won many gallantry awards and military honors. Gorkha was the usual designation of the reigning dynasty of Nepaul (Nepal)^1^ and the designation had no ethnic connotation^2^. Historically, the terms ‘*Gurkha*’ and ‘*Gurkhali*’ are synonymous with ‘*Nepali*’ and were derived from the name of old kingdom of Gorkha (Gurkha), a hill town and present day district of Gorkha (~fifty kilometers west of Kathmandu) from which the kingdom of Nepal expanded^3^. As the kingdom spread across the Himalayas from Tibet to Sikkim, the king’s warriors, taken from all groups in the area, came to be known as Gorkha soldiers^2^. During the Gurkha War (1814–1816) between the Gorkha Kingdom and the East India Company, the British were impressed by the Gorkhali soldiers whom they called Gurkhas^4^ and they became a part of the British Indian Army. After India’s independence in 1947, as part of the Tripartite Agreement, the Gurkha Regiment was split between the British and the Indian Army. There are two types of Gorkhas in the Indian Army: the original inhabitants of Nepal and the ones who are domiciled in India (who migrated from Nepal long ago and settled in the hilly region of Northern and North-East India).

Only a handful of published genetic studies on Gorkhas/Nepalese are available^5^,^6^,^7^,^8^,^9^,^10^,^11^,^12^. Nepal is situated just to the south of the Himalayan mountain peaks. Himalaya, stretching from Pakistan to Myanmar, forms the highest land boundary on our planet and is one of the linguistically most complex regions. The Great Himalayan region is of particular interest for studying human population prehistory. The geographical area comprising the present day Nepal provided corridors for human migration in ancient times, resulting in relatively early inhabitation of the area. Nepal is a multiethnic, multilingual and multicultural country made up of more than 125 ethnic/caste groups and more than 123 languages^13^ which are derived mostly from three major language groups: Indo-Aryan, Tibeto-Burman and various indigenous language isolates. The two major groups in the Nepali society are Tibeto-Burman or Mongoloids from the north and the Indo-Aryans from the south. The larger groups can be divided on the basis of geographical locations by altitude: alpine based cultural groups (Sherpas, Dolpas, Larkes and Siars, Manang bas, Lo pas, Olangchung), temperate zone based cultural groups (Brahmins and Chhetris, Kiratis, Tamangs, Magars, Gurungs, Thakalis) and subtropical based cultural groups (Brahmans and Rajputs, Tharus, Rajbansis, Satars, Musalmans)^14^. It is generally believed that ancestors of ethnic groups inhabiting the northern Nepal, the middle hills and the north eastern region (Gurungs, Magars, Limbus, Rais, Sherpas, Tamangs and Thakalis etc.) came from Tibet, China, Myanmar, Mongolia and the Far East. Majority of Gurungs along with Magars and their Khasa counterparts formed the bulk of the famous Gorkha regiment of British and Indian Army.

Nepalese population displays strong genetic differentiation despite sharing close geographical proximity underscoring necessity for studies of Nepalese ethnic groups. The understanding of past genetic history, genetic affinity as well as population diversity has been vastly improved by the molecular genetic markers like microsatellite, mitochondrial DNA and Y chromosome markers. Microsatellite markers, particularly the autosomal short tandem repeats (STR), which have high polymorphic information content (PIC) and high power of discrimination^15^, have become the markers of choice for genetic analysis and understanding of genetic relatedness of the population. The present study investigates i) the genetic status of the Gorkhas of Tibeto-Burman linguistic phyla serving the Indian Army based on a set of 15 autosomal microsatellite (STR) markers, ii) the extent of affiliation with ethno-linguistically close population of Nepal, Tibet and other global Asian populations belonging to the Tibeto-Burman linguistic family and iii) construction of Indian soldier DNA dataset for human identification for military purposes.

## Results and Discussion

In total, 138 alleles were observed in the studied population with allele frequencies ranging from 0.005 to 0.469. *TPOX* locus had the maximum allele frequency with allele 8 (0.469) being the most frequent allele in the population. Observed heterozygosity of *TPOX, TH01* and *D5S818* was found to be low being 0.602, 0.663 and 0.663 respectively. Remaining STR loci were highly polymorphic with observed heterozygosity values ranging from 0.724 for *CSF1PO* to 0.867 for *D18S51*. All loci met HWE expectations. Allele frequencies and summary statistics of the 15 autosomal STRs in the studied Gorkhas are presented in Table 1. Polymorphism Information Content (PIC) in the population ranged from 0.61 (*TPOX*) to 0.87 (*FGA*) and power of discrimination (PD) ranged from a minimum of 0.843 for *TPOX* to a maximum of 0.963 for *FGA*. The most polymorphic locus of *FGA* was also, as was expected, the most discriminating in the population. The power of exclusion (PE) ranged from a minimum of 0.293 for *TPOX* to a maximum of 0.770 for *D21S11* and *D19S433*. The combined power of discrimination (CPD) and combined power of exclusion (CPE) for all 15 STR loci was 0.9999999986 and 0.9999993441 respectively. The average probability of matching value was found to be 0.082 and it was expressed as 1 in 14.7.

**Figure 1:**
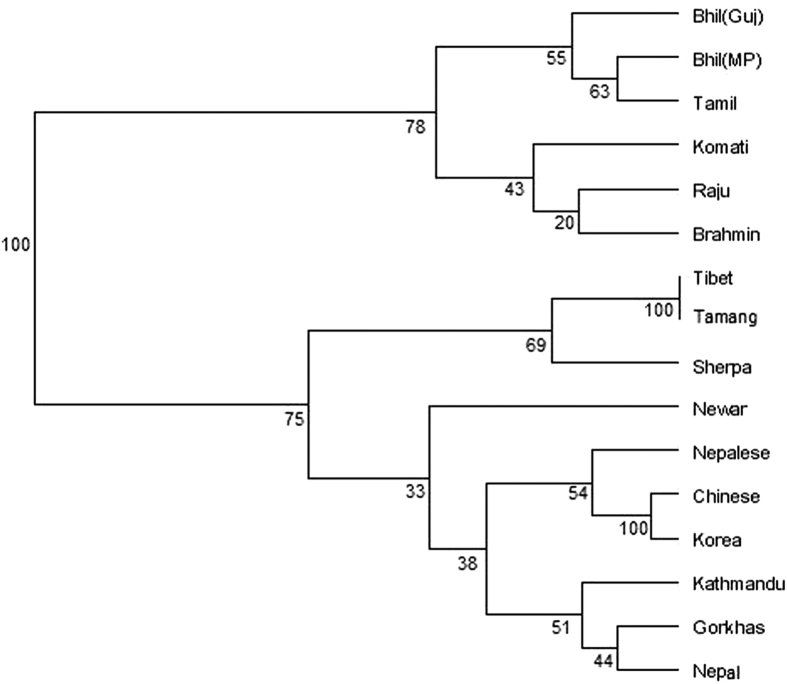
Neighbor Joining Tree based on Nei’s genetic distance showing the genetic relationship of Gorkhas with other neighboring population groups and lowlander Indians based on 15 STR markers.

**Figure 2:**
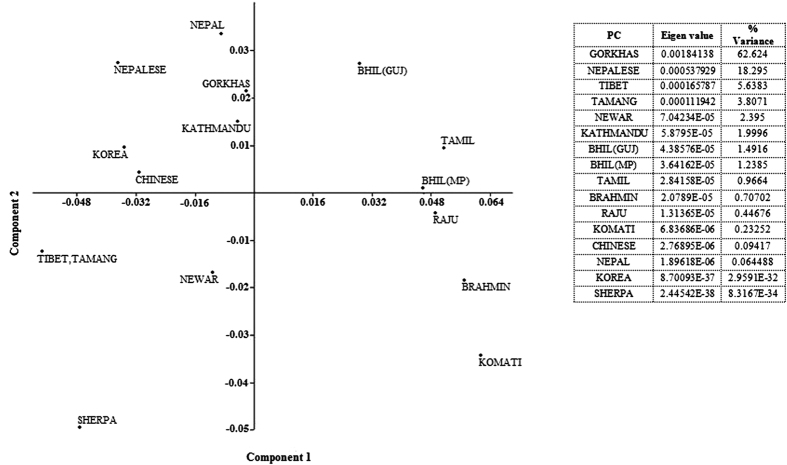
Principal Component Analysis (PCA) plot showing distance pattern of Gorkhas with other published population based on Nei’s Da distance matrix.

**Figure 3:**
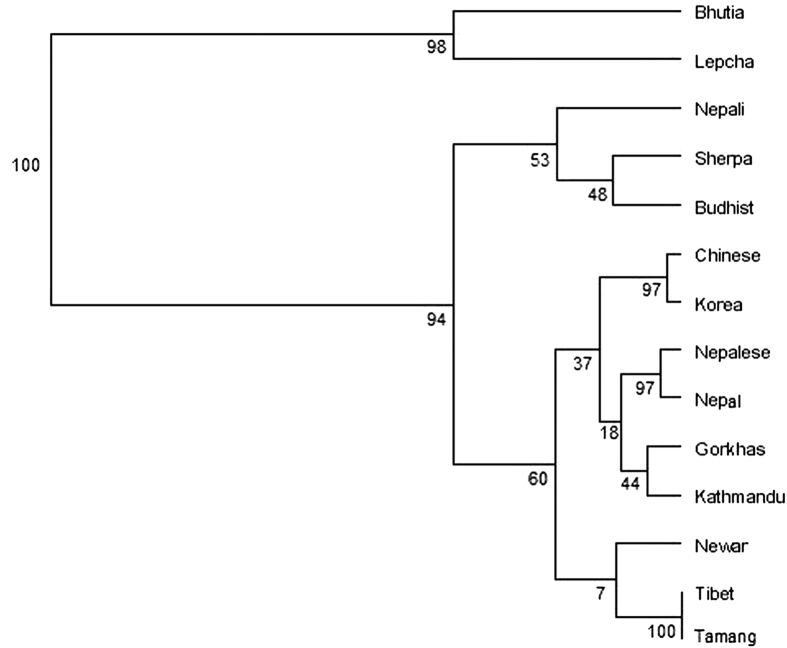
Neighbor Joining Tree showing genetic relationship of Gorkhas with high altitude native (Buddhist) of Ladakh and other neighboring population based on 9 STR markers.

Phylogenetic analysis based on allelic frequencies was performed to investigate genetic relationship of the studied population with the neighboring and Indian lowlander population^8^,^9^,^10^,^16^,^17^,^18^,^19^,^20^,^21^,^22^ using a set of 15 STR loci. Fst values ranged from (−) 0.00645 to 0.21675 (Supplementary Table 1). Locus-wise comparison of allele frequencies and pair wise Fst p value is shown in Table 2. The pair wise Fst values showed significant similarity at all 15 loci with Nepal population^19^ and at 14 loci with Nepalese population^8^, Tibeto-Burman speaking Tamang^10^ and Indo-European (Nepali) speaking people from Kathmandu^10^ (Supplementary Table 1). Allele frequencies at 11 out of the 15 loci in the Gorkhas were statistically similar to the Tibetan general population^10^. Interestingly, Sherpas, a Tibetan population from Namche Bazaar, Nepal^9^, situated at an elevation of 3440 m, showed similarity with the Gorkhas at only 9 out of 15 loci in the present study (Supplementary Table 1). This observation is not unexpected as in an earlier study, allelic and genotypic distributions between Sherpas of Namche Bazaar and non-Sherpas from Kathmandu valley were shown to differ significantly at 14 STR loci^9^. The Nei’s D_A_ distance matrix is presented in Table 3. Geographically close Gorkha population (present study) and those from Nepal^8^,^19^ showed the minimum distance value reflecting closeness. Phylogenetic trees established with the genetic distance matrix constructed with allele frequency information of 15 STR markers showed clustering of the Gorkhas with the population from Nepal^19^, Kathmandu^10^ and other Tibeto-Burman linguistic phyla as one group while the lowlander Indian population formed a separate cluster (Fig. 1). Figure 2 shows the PCA plot based on Component I and Component II scores. In general, the scattering pattern obtained in PCA (Fig. 2) showed similarity with clustering pattern of NJ tree (Fig. 1). Four separate aggregates were evident in PCA plot: Indian lowlanders comprising Bhils and Tamils were placed in the upper right quadrant, Indian lowlanders comprising Brahmins, Komatis and Rajus were placed in the lower right quadrant, Gorkhas of present study were placed in the upper left quadrant along with Nepalese^8^, Kathmandu^10^, Nepal^19^ as well as with Korean^18^ and Chinese^20^ population. It is worthwhile to mention here that low bootstrap value between Gorkhas and Nepalese was observed in the phylogenetic tree (Fig. 1) created by software POPTREE2^23^. Nevertheless, further estimation of relatedness between the two populations by pair wise Fst values computed by Arlequin version 3.5 software^24^ (Table 2), Nei’s DA distance matrix obtained by POPTREE2^23^ (Table 3) as well as placement of populations in PCA plot created by Past software package^25^ (Fig. 2) substantiated the relatedness of the studied populations. Neighbor Joining dendogram constructed from Nepali speaking community from Sikkim along with the tribal population of Lepchas and Bhutias^26^ and high altitude native population from Ladakh, particularly the Buddhists^27^ (who also belong to Tibeto-Burman linguistic phyla) based on allele frequency information from 9 STR markers, did not cluster the Gorkhas with high altitude native (Buddhist) population from Ladakh or with Lepchas and Bhutias or Nepali speaking community of Sikkim (Fig. 3), which in all probability may reflect separate migration history.

The present study reveals phylogenetic relationship of Gorkhas of Tibeto-Mongol origin with neighboring Himalayan population. Our findings are in concordance with the previous studies on Indian Gorkhas which suggested genetic closeness of Gorkha population with that of Mongoloid origin based on HLA-A and B antigens^7^. A characteristic HLA A33-HLA B44 haplotype of Korean and Japanese population was shown to occur with significant positive association in Gorkhas^7^. An earlier study by Wang and colleagues^11^ revealed that vast majority of Nepali gene pool comprised genetic component of East Eurasian (36.56%) and South Asian (51.63%) ancestry and 3 out of 5 Nepalese populations were clustered with the Tibetan population. The Himalayan region of the Indian subcontinent contains a complex linguistic pattern indicative of the region being an ancient source of genetically differentiated population and language^28^. The Himalayan mountain ranges separate the Tibetan plateau from the Indian subcontinent forming a linguistic boundary between the Tibeto-Burman and Indo-European language families^29^; language and geography might have played equally important roles in defining the genetic composition of present day Himalayan population^28^.

Geographical continuity is a major influencing factor of genetic affinity among diverse populations which are distributed over a wide geographical area after migration. Geographically adjoining populations from a common stock cluster together due to genetic affinity and the findings of the present study with autosomal STR markers substantiate the genetic affinity of the Gorkhas with the Tibeto-Burman linguistic phyla reflecting recent past genetic history and possible migration from Tibet as well as probable origin of the Gorkhas from Mongolian and/or Tibetan stocks. Studies with Y- chromosomal diversity revealed high frequency of East Asian specific haplogroup O3a5-M1324 in the Himalayan population of Nepal and Tibet suggesting a common ancestry for these linguistic subfamilies^30^. Higher prevalence of South Asian-derived Y-haplogroup R1a1-M198 was also reported in the Nepalese population of Newar and Kathmandu indicating significant genetic influence from the Indian subcontinent^30^. Previous studies had strongly suggested that most of the East Eurasian maternal components identified in Nepalese were directly introduced from Tibet^8^,^28^. A maternal footprint of gene flow was reported between Nepal and India^31^.

**Figure 4:**
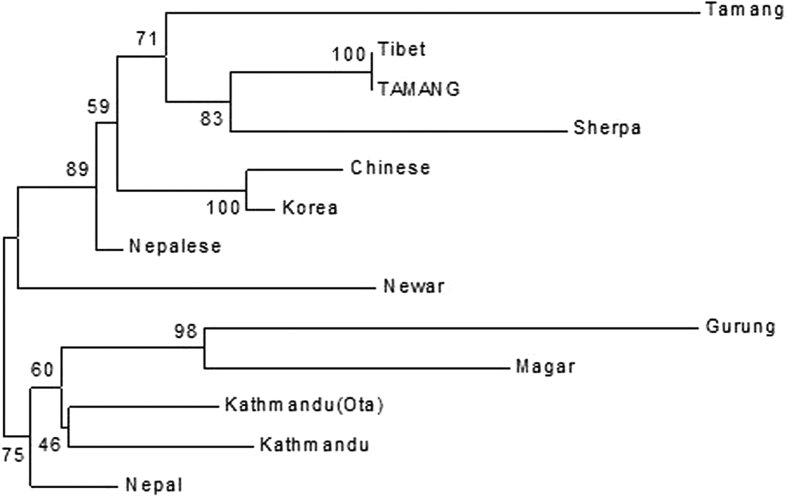
Neighbor Joining Tree showing genetic relationship of Tamangic (Gurung, n = 33 and Tamang, n = 33) and Magaric (Magar, n = 34) with other neighboring population based on 15 STR markers.

It was observed that our study population comprised both Tamangic (Gurung, Tamang) and Magaric (Magar) groups of Tibeto-Burman language family, based on assessment of ethnicity from ethno-linguistic questionnaire (Table 4). Further investigation of phylogenetic relationship between the Gurung, Tamang and Magar groups showed clustering of the Tamangs with Tibetans^10^, Tamangs^10^ and Sherpas^9^ while Gurungs and Magars showed genetic relatedness with those from Kathmandu, Nepal^9^,^10^ (Fig. 4). Gurungs and Magars were also closely clustered suggesting common origin of these two ethnic groups (Fig. 4). This interesting observation, however, is required to be substantiated by increasing the markers and the ethnic groups. Although little is known about Tamang history, it is believed that they came from Tibet possibly around 3000 years ago. The Magar people (genetically and physically Mongoloid/East Asian) are believed to have migrated from Tibet via Sikkim although their origin is shrouded in mystery. Origin of Gurungs is also uncertain though linguistic evidence suggests that their ancestors may have migrated from Tibet about 2000 year ago. They are predominantly of Mongoloid racial stock and speak a language which largely belongs to the Tibeto-Burman language family^32^. Time estimation results indicate that people from Tibet began to migrate to Nepal around 6000 years ago^11^ which is also in agreement with the archeological findings of reported sharing of Neolithic features between Nepal and Tibet^33^ and historically recorded passes (Kodari and Rasuwa) which had connected the Nepalese and the Tibetans since the ancient times^34^. A recent study has revealed presence of Denisovan haplotype in the Himalayan population^35^.

India is home to various social groups with diverse ethnic and linguistic origins representing several racial stocks and social statuses that found place for themselves at various points of time and adapted to several ecological niches offered by the physiographic and climatic setting of the area. The waves of migration drew the ancestors of the majority of the present population of the area from the surrounding territories and across the Himalayas^36^. India, with diverse human population, provides a unique opportunity for population genetics explorations. Tibeto-Burman speaking population, which is one of the four major linguistic groups in India, differs from other linguistic families of India and contributes to the component of diversity to peopling of India. Very few studies are available addressing the extent of genetic diversity and genetic affinity in the Tibeto-Burman population^37^,^38^,^39^,^40^,^41^. The present study with autosomal STR markers is the first study conducted on Tibeto-Burman speaking Gorkhas from the Indian Armed Forces and substantiates the genetic affinity of the Gorkhas with the Tibeto-Burman linguistic phyla. The results demonstrate that geographic isolation has not played significant role in differentiation of genetic constitution of the Gorkhas whether they come from Nepal or are domiciled in India.

One of the other objectives of our study was to create a microsatellite dataset of Indian soldiers for human identification for military purposes. Military exigencies and major disasters such as wars, airplane accidents and maritime transport disasters leave military personnel highly vulnerable. Usage of weapons of mass destruction and terrorist action also expose the military to disaster and fatality. Identification of remains brings sense of comfort and closure to family and friends. The STR markers are important tools for human identification^18^,^42^. Various countries have national forensic DNA databases: the Combined DNA Index System (CODIS) in USA, the National DNA database (NDNAD) in UK and Fichier National Automatisé des Emprintes Génétiques (FNAEG) in France which is used by the national police force as well as the military police. Indian Armed Forces has initiated a project of DNA profiling of military personnel which is expected to be completed by 2020. The dataset of STR markers obtained in this study presents a valuable information source of STR DNA profiles from military personnel for usage in disaster victim identification in military exigencies. There is also an urgent need to formulate the DNA profiling laws in India.

## Conclusion

The present study reveals phylogenetic relationship of Gorkhas of Tibeto-Mongol origin with other neighboring Tibeto-Mongol Himalayan population. The study substantiates genetic affinity of the Gorkhas with the Tibeto-Burman linguistic phyla reflecting recent past genetic history and origin of the Gorkhas from Mongolian and/or Tibetan stocks. Furthermore, the dataset of STR markers obtained in this study presents a valuable information source of STR DNA profiles from military personnel for usage in disaster victim identification in military exigencies and adds to the Indian database of military soldiers and military hospital repository.

## Methods

The study and the experimental protocols were approved by the Ethics Committee of the Defence Institute of Physiology and Allied Sciences, Delhi. The participants (n = 100) were selected from the Gorkha regiment of the Indian Army who were part of an ongoing study of the Institute. All participants gave written informed consent before enrolment in the study. The experiments were conducted in accordance with quality control measures at the Department of Molecular Biology of Defence Institute of Physiology and Allied Sciences, Delhi, Ministry of Defence, India which is an accredited laboratory (ISO 9001:2008).

### Participants and sample collection

Of the participants who reported, 59 individuals were from Nepal and 41 from India. During the study period, they were stationed at sea level. The clan ties were determined based on self-reporting and ethnic backgrounds were ascertained through ethno-linguistic questionnaire. To ensure that the individuals were ethnically unmixed, both parents had to belong to the same group. 3–4 ml of peripheral blood was collected through venepuncture from the consenting individuals in K_2_ EDTA vacutainers (BD, CA, USA) and stored at −20 °C till further processing.

### DNA extraction and microsatellite typing

High molecular genomic DNA was extracted as per published procedure^43^. Quantity assessment was performed on Nanodrop 2000C (Thermo Fisher, USA) and quality checked by Agarose gel electrophoresis. DNA samples had a A_260_/A_280_ ratio of 1.8–1.9. Further quantification of DNA was performed by the Quantifiler Duo Human DNA Quantification kit (Applied Biosystems, Foster City, CA) using 7500 Real Time PCR system (Applied Biosystems, Foster City, CA) with v1.1 software as per manufacturer’s protocol.

Polymerase chain reaction (PCR) amplification was carried out for each sample using 15 autosomal STR loci markers (*D8S1179, D21S11, D7S820, CSF1PO, D3S1358, TH01, D13S317, D16S539, D2S1338, D19S433, vWA, TPOX, D18S51, D5S818* and *FGA*) along with the gender determination marker *Amelogenin* with AmpFlSTR^®^ Identifiler^®^ Plus kit (Applied Biosystems, Foster City, CA) on GeneAmp PCR system 9700 Thermal Cycler following manufacturer’s recommended protocol. 2 samples failed in amplification. Positive and negative amplification controls were used as per kit guidelines. The amplified products were run on 3500xL Genetic Analyzer (Applied Biosystems, Foster City, CA) using 36 cm capillary array and Dye set G5. Allelic ladder sample provided in the kit was included in each run. Data generated using capillary electrophoresis was analyzed using Gene Mapper ID-X version 1.4 software (Applied Biosystems, Foster City, CA) as per manufacturer’s instructions. Allele calls were generated for all samples and exported in Excel format. Plot views were generated in PDF format.

### Analytical methods

Allelic frequencies for the 15 STR loci and matching probability (Pm), power of discrimination (PD), power of exclusion (PE) and polymorphic information content (PIC) were computed using the PowerStats version 1.2 spreadsheet program^44^. Arlequin^24^ version 3.5 was used to calculate observed (H_obs_) and expected heterozygosity (H_exp_) and Hardy-Weinberg Equilibrium (HWE). HWE based on the exact test was confirmed for all the studied 15 loci at a significance level of p >0.003 after Bonferroni correction^45^ (α = 0.05/15 = 0.003). In the absence of raw genotypic scores from other populations, published allele frequency datasets of STR loci from neighboring populations were used for population differentiation by Arlequin using Fst pair wise distance. Phylogenetic analysis based on allele frequencies were performed to investigate the genetic relationship between the Gorkha population, other neighboring population and Indian lowlanders using the set of 15 STR loci and 9 STR loci from different datasets. POPTREE2 software^23^ was used for generating Neighbor Joining (NJ) dendograms as well as to derive Nei’s genetic distances^46^. Robustness of the phylograms established by NJ tree was estimated by bootstrapping 1000 replicates over loci. Principal Component Analysis (PCA) plot was generated with Past software package^25^ version 3.02 and used for graphical representation of the genetic distances (Dst) of the Gorkha population with other global/Indian lowlander populations.

## Additional Information

**How to cite this article**: Preet, K. *et al.* Genetic diversity in Gorkhas: An autosomal STR study. *Sci. Rep.* **6**, 32494; doi: 10.1038/srep32494 (2016).

## Supplementary Material

Supplementary Information

## Figures and Tables

**Table 1:** Observed allele frequency for 15 autosomal STR loci in Gorkha population (n = 98).

Allele	D8S1179	D21S11	D7S820	CSF1PO	D3S1358	TH01	D13S317	D16S539	D2S13338	D19S433	vWA	TPOX	D18S51	D5S818	FGA
6						0.153									
7			0.026			0.204								0.005	
8	0.005		0.189	0.010		0.107	0.189	0.066				0.469			
9			0.061	0.046		0.418	0.133	0.230				0.122		0.046	
9.1			0.005												
9.3						0.107									
10	0.117		0.158	0.194		0.010	0.102	0.046			0.005	0.082		0.143	
11	0.071		0.276	0.250			0.245	0.270		0.010		0.306	0.031	0.337	
11.2										0.010					
12	0.112		0.260	0.434			0.276	0.235		0.036		0.020	0.046	0.332	
12.2										0.010					
13	0.168		0.015	0.061			0.046	0.122		0.256			0.158	0.128	
13.2										0.026					
14	0.179		0.010	0.005	0.041		0.010	0.031		0.316	0.138		0.199	0.010	
14.2										0.082					
15	0.255				0.321				0.005	0.092	0.082		0.214		
15.2										0.092					
16	0.082				0.265				0.056	0.041	0.230		0.087		
16.2										0.010					
17	0.005				0.245				0.133		0.230		0.066		
17.2									0.189	0.010					
18	0.005				0.117				0.077		0.199		0.036		0.005
19					0.005						0.102		0.087		0.128
20					0.005				0.005		0.015		0.041		0.071
20.2									0.071						0.005
21													0.015		0.107
22									0.138						0.138
22.2															0.010
23									0.199				0.005		0.153
23.2															0.005
24									0.097				0.010		0.153
24.2									0.031						0.005
25													0.005		0.138
26															0.046
27															0.026
28		0.133													0.005
29		0.214													0.005
29.2		0.010													
30		0.230													
30.2		0.036													
31		0.082													
31.2		0.107													
32		0.005													
32.2		0.122													
33.2		0.046													
34.2		0.015													
PD	0.944	0.950	0.918	0.863	0.888	0.887	0.923	0.927	0.960	0.924	0.931	0.843	0.957	0.885	0.963
PIC	0.820	0.830	0.760	0.660	0.710	0.700	0.770	0.770	0.850	0.780	0.790	0.610	0.850	0.690	0.870
PE	0.649	0.770	0.573	0.467	0.519	0.536	0.591	0.649	0.630	0.770	0.649	0.293	0.729	0.374	0.689
PI	2.880	4.450	2.330	1.810	2.040	2.130	2.450	2.880	2.720	4.450	2.880	1.260	3.770	1.480	3.270
H_obs_	0.827	0.857	0.786	0.724	0.755	0.663	0.796	0.827	0.816	0.827	0.827	0.602	0.867	0.663	0.847
H_exp_	0.841	0.812	0.794	0.709	0.755	0.651	0.802	0.801	0.868	0.722	0.823	0.667	0.868	0.742	0.877
p value	0.058	0.779	0.680	0.504	0.412	0.707	0.162	0.970	0.186	0.074	0.117	0.736	0.059	0.057	0.153
Pm	0.056	0.050	0.082	0.137	0.112	0.113	0.077	0.073	0.040	0.076	0.069	0.157	0.043	0.115	0.037

PD, Power of discrimination; PIC, Polymorphism information content; PE, Power of exclusion; PI, Paternity index; H_obs_, Observed heterozygosity; H_exp_, Expected heterozygosity; p value HWE test ; Pm, Matching probability.

**Table 2:** Population differentiation Fst p-values resulting from the locus-wise comparison of Gorkhas with nine neighboring population and six lowlander Indian population.

Gorkha vs…	n	n sign.	D8S1179	D21S11	D7S820	CSF1PO	D3S1358	TH01	D13S317	D16S539	D2S1338	D19S433	vWA	TPOX	D18S51	D5S818	FGA
Nepal^19^	233	0	0.450	0.694	0.216	0.396	0.063	0.486	0.820	0.207	0.180	0.207	0.613	0.297	0.171	0.135	0.198
Nepalese^8^	953	1	0.036	0.586	0.622	0.577	0.027	0.036	0.027	0.036	0.117	0.387	0.126	0.270	0.054	**0.000**	0.036
Tamang^10^	45	1	0.694	0.937	0.901	0.91	0.306	0.387	0.117	0.261	0.018	0.441	0.685	0.045	**0.000**	0.054	0.351
Kathmandu^10^	77	1	0.171	0.883	0.315	0.523	0.009	0.477	0.09	**0.000**	0.297	0.477	0.748	0.252	0.279	0.279	0.126
Newar^10^	66	2	0.378	0.216	0.189	0.342	0.090	0.171	0.270	0.568	0.027	**0.000**	0.36	0.396	**0.000**	0.099	0.027
Sherpa^9^	105	6	0.045	0.441	0.018	0.550	**0.000**	0.009	**0.000**	0.189	**0.000**	0.045	0.135	0.153	**0.000**	**0.000**	**0.000**
Tibet^10^	153	4	0.369	0.595	0.649	0.793	0.090	0.081	0.027	0.072	**0.000**	0.117	0.351	**0.000**	**0.000**	**0.000**	0.063
Chinese^20^	1161	5	0.009	**0.000**	**0.000**	0.324	0.243	0.018	**0.000**	0.027	0.045	0.189	**0.000**	0.054	0.126	**0.000**	0.009
Korea^18^	1805	8	**0.000**	**0.000**	0.045	**0.000**	0.027	0.108	**0.000**	**0.000**	**0.000**	0.099	**0.000**	0.324	0.009	0.009	**0.000**
Bhil (Guj)^21^	297	4	0.658	0.027	0.018	0.378	0.928	0.009	0.234	0.540	**0.000**	0.009	**0.000**	**0.000**	**0.000**	0.486	0.108
Bhil (MP)^22^	183	8	0.523	0.009	**0.000**	**0.000**	0.243	**0.000**	0.477	**0.000**	**0.000**	0.045	0.297	**0.000**	**0.000**	0.306	**0.000**
Tamil^17^	272	7	**0.000**	0.009	0.018	0.045	0.667	**0.000**	0.162	**0.000**	**0.000**	**0.000**	0.18	**0.000**	0.108	0.198	**0.000**
Brahmin^16^	106	5	0.279	0.045	**0.000**	0.162	0.198	0.018	0.279	0.045	**0.000**	0.810	0.459	**0.000**	**0.000**	0.342	**0.000**
Raju^16^	66	2	0.027	0.702	0.009	0.234	0.45	0.180	0.468	0.027	0.009	0.045	0.072	**0.000**	0.063	0.243	**0.000**
Komati^16^	104	9	0.288	**0.000**	**0.000**	0.099	0.261	**0.000**	**0.000**	**0.000**	**0.000**	0.441	**0.000**	0.063	**0.000**	**0.000**	0.027

n, number of individuals in the population; n sign., number of markers showing significant p-values (**in bold**). p < 0.003, statistically significant.

**Table 3:** Matrix of Nei’s D_A_ distance between pairs of population studied.

	Gorkha	Nepalese	Tibet	Tamang	Newar	Kathmandu	Bhil (Guj)	Bhil (MP)	Tamil	Brahmin	Raju	Komati	Chinese	Nepal	Korea	Sherpa
Gorkha	0.000															
Nepalese^8^	0.015	0.000														
Tibet^10^	0.027	0.012	0.000													
Tamang^10^	0.027	0.012	0.000	0.000												
Newar^10^	0.032	0.021	0.033	0.033	0.000											
Kathmandu^10^	0.023	0.016	0.029	0.029	0.029	0.000										
Bhil (Guj)^21^	0.02	0.022	0.035	0.035	0.029	0.022	0.000									
Bhil (MP)^22^	0.028	0.033	0.047	0.047	0.039	0.030	0.012	0.000								
Tamil^17^	0.025	0.033	0.047	0.047	0.042	0.026	0.014	0.017	0.000							
Brahmin^16^	0.032	0.038	0.053	0.053	0.053	0.036	0.024	0.033	0.023	0.000						
Raju^16^	0.029	0.032	0.050	0.050	0.042	0.033	0.022	0.027	0.021	0.030	0.000					
Komati^16^	0.038	0.043	0.057	0.057	0.047	0.044	0.026	0.033	0.029	0.032	0.028	0.000				
Chinese^20^	0.023	0.013	0.021	0.021	0.036	0.028	0.030	0.042	0.039	0.042	0.038	0.044	0.000			
Nepal^19^	0.016	0.010	0.022	0.022	0.024	0.015	0.016	0.025	0.022	0.030	0.027	0.036	0.020	0.000		
Korea^18^	0.021	0.011	0.019	0.019	0.031	0.026	0.029	0.040	0.037	0.043	0.036	0.044	0.006	0.018	0.000	
Sherpa^9^	0.038	0.023	0.023	0.023	0.041	0.036	0.044	0.051	0.055	0.062	0.057	0.061	0.033	0.033	0.027	0.000

**Table 4:** Details of participants based on ethno linguistic grouping.

Group	Region of Origin	Linguistic phylum*	n, males
Gurung	Nepal	TB, Tamangic	17
Gurung	India	TB, Tamangic	16
Tamang	Nepal	TB, Tamangic	16
Tamang	India	TB, Tamangic	17
Magar	Nepal	TB, Magaric	26
Magar	India	TB, Magaric	08

*Classification based on van Driem (2001); n, number.
