# Supplementary material for: Genetic Diversity in Gorkhas: an Autosomal STR Study
Source: Sci Rep. 2016 Sep 1;6:32494. doi: 10.1038/srep32494 (PMC5007509; doi:10.1038/srep32494)
Supplement: Supplementary Information [file srep32494-s1.pdf]

GENETIC DIVERSITY IN GORKHAS: AN AUTOSOMAL STR STUDY
Kiran Preet1, Seema Malhotra1, Pankaj Shrivastava2, Toshi Jain2, Shweta Rawat1, L Robert Varte1, Sayar Singh1, Inderjeet Singh1, Soma Sarkar1*
1Defence Institute of Physiology and Allied Sciences, DRDO, Lucknow Road, Timarpur, Delhi-110054, India. 2State Forensic Science Laboratory, Civil Lines, Sagar, Madhya Pradesh-470001, India.

Supplementary Table 1: Population differentiation Fst values of Gorkhas with other Neighboring population and Indian lowlanders

Gorkhas	Tibet	Tamang	Newar	Kathmandu	Nepalese	Bhil(Guj)	Bhil(MP)	Tamil	Brahmin(AP)	Raju	Komati	Chinese	Korea	Nepal	Sherpa
D8S1179															
Fst	0.00087	-0.00305	0.00008	0.00429	0.00274	-0.00098	-0.00033	0.04605	0.00099	0.01013	0.00128	0.00611	0.0069	-0.00004	0.00647
P value	0.36937+-0.0344	0.69369+-0.0238	0.37838+-0.0556	0.17117+-0.0316	0.03604+-0.0148	0.65766+-0.0305	0.52252+-0.0354	0.00000+-0.0000	0.27928+-0.0550	0.02703+-0.0139	0.28829+-0.0485	0.00901+-0.0091	0.00000+-0.0000	0.45045+-0.0650	0.04505+-0.0203
D21S11															
Fst	-0.00125	-0.00603	0.00189	-0.00436	-0.00104	0.00502	0.00994	0.00739	0.0044	-0.00278	0.0298	0.01166	0.00954	-0.00175	0.00013
P value	0.59459+-0.0511	0.93694+-0.0203	0.21622+-0.0364	0.88288+-0.0184	0.58559+-0.0455	0.02703+-0.0139	0.00901+-0.0091	0.00901+-0.0091	0.04505+-0.0203	0.70270+-0.0572	0.00000+-0.0000	0.00000+-0.0000	0.00000+-0.0000	0.69369+-0.0430	0.44144+-0.0252
D7S820															
Fst	-0.00185	-0.0055	0.00383	0.0011	-0.00088	0.00794	0.01115	0.00683	0.04398	0.01126	0.02583	0.02055	0.0057	0.00118	0.00861
P value	0.64865+-0.0425	0.90090+-0.0304	0.18919+-0.0438	0.31532+-0.0412	0.62162+-0.0515	0.01802+-0.0121	0.00000+-0.0000	0.01802+-0.0121	0.00000+-0.0000	0.00901+-0.0091	0.00000+-0.0000	0.00000+-0.0000	0.04505+-0.0203	0.21622+-0.0473	0.01802+-0.0182
CSF1PO															
Fst	-0.00247	-0.00645	0.00046	-0.00195	-0.00106	0.0003	0.03936	0.00664	0.00302	0.00171	0.00545	0.00109	0.11575	0.00004	-0.00169
P value	0.79279+-0.0354	0.90991+-0.0253	0.34234+-0.0327	0.52252+-0.0297	0.57658+-0.0609	0.37838+-0.0402	0.00000+-0.0000	0.04505+-0.0152	0.16216+-0.0264	0.23423+-0.0364	0.09910+-0.0286	0.32432+-0.0528	0.00000+-0.0000	0.39640+-0.0511	0.54955+-0.0370
D3S1358															
Fst	0.00544	0.00167	0.00633	0.0144	0.00432	-0.00273	0.00112	-0.00152	0.00142	-0.00011	0.00132	0.00033	0.00526	0.00453	0.01955
P value	0.09009+-0.0235	0.30631+-0.0433	0.09009+-0.0303	0.00901+-0.0091	0.02703+-0.0194	0.92793+-0.0238	0.24324+-0.0360	0.66667+-0.0310	0.19820+-0.0264	0.45045+-0.0507	0.26126+-0.0316	0.24324+-0.0451	0.02703+-0.0139	0.06306+-0.0237	0.00000+-0.0000
TH01															
Fst	0.00495	0.00141	0.00461	-0.00129	0.0041	0.01046	0.02089	0.02831	0.01108	0.00459	0.02466	0.00761	0.00238	-0.00059	0.01402
P value	0.08108+-0.0212	0.38739+-0.0430	0.17117+-0.0286	0.47748+-0.0508	0.03604+-0.0201	0.00901+-0.0091	0.00000+-0.0000	0.00000+-0.0000	0.01802+-0.0121	0.18018+-0.0489	0.00000+-0.0000	0.01802+-0.0121	0.10811+-0.0326	0.48649+-0.0364	0.00901+-0.0091
D13S317															
Fst	0.01061	0.00711	0.00132	0.00583	0.00448	0.00125	-0.00041	0.0021	0.0008	-0.00012	0.15152	0.01858	0.00918	-0.00186	0.03269
P value	0.02703+-0.0139	0.11712+-0.0237	0.27027+-0.0407	0.09009+-0.0303	0.02703+-0.0139	0.23423+-0.0411	0.47748+-0.0705	0.16216+-0.0379	0.27928+-0.0497	0.46847+-0.0556	0.00000+-0.0000	0.00000+-0.0000	0.00000+-0.0000	0.81982+-0.0253	0.00000+-0.0000
D16S539															
Fst	0.0059	0.00209	-0.00215	0.0197	0.00545	-0.00056	0.01018	0.01067	0.00652	0.01152	0.01887	0.00509	0.01121	0.00122	0.00154
P value	0.07207+-0.0264	0.26126+-0.0344	0.56757+-0.0526	0.00000+-0.0000	0.03604+-0.0148	0.54054+-0.0192	0.00000+-0.0000	0.00000+-0.0000	0.04505+-0.0203	0.02703+-0.0139	0.00000+-0.0000	0.02703+-0.0139	0.00000+-0.0000	0.20721+-0.0305	0.18919+-0.0394
D2S1338															
Fst	0.01269	0.00896	0.00633	0.00099	0.00203	0.00616	0.00988	0.02004	0.01184	0.00919	0.02387	0.00291	0.03359	0.00105	0.01389
P value	0.00000+-0.0000	0.01802+-0.0121	0.02703+-0.0139	0.29730+-0.0490	0.11712+-0.0333	0.00000+-0.0000	0.00000+-0.0000	0.00000+-0.0000	0.00000+-0.0000	0.00901+-0.0091	0.00000+-0.0000	0.04505+-0.0203	0.00000+-0.0000	0.18018+-0.0332	0.00000+-0.0000
D19S433															
Fst	0.0032	-0.00153	0.03301	-0.00164	-0.00008	0.00744	0.00745	0.0106	-0.00335	0.01114	0.00005	0.00148	0.00183	0.00062	0.00987
P value	0.11712+-0.0273	0.44144+-0.0317	0.00000+-0.0000	0.47748+-0.0385	0.38739+-0.0430	0.00901+-0.0091	0.04505+-0.0152	0.00000+-0.0000	0.81081+-0.0304	0.04505+-0.0152	0.44144+-0.0497	0.18919+-0.0438	0.09910+-0.0212	0.20721+-0.0592	0.04505+-0.0152
vWA															
Fst	0.00015	0.00356	0.00077	-0.00299	0.00198	0.01799	0.00132	0.00161	-0.0006	0.0049	0.02593	0.0166	0.00662	-0.00119	0.00218
P value	0.35135+-0.0370	0.68468+-0.0310	0.36036+-0.0303	0.74775+-0.0361	0.12613+-0.0278	0.00000+-0.0000	0.29730+-0.0451	0.18018+-0.0332	0.45946+-0.0684	0.07207+-0.0227	0.00000+-0.0000	0.00000+-0.0000	0.00000+-0.0000	0.61261+-0.0379	0.13514+-0.0412
TPOX															
Fst	0.02811	0.02298	0.00125	0.00154	0.00032	0.03271	0.05836	0.04969	0.02881	0.06692	0.00906	0.00455	0.00037	0.00019	0.00586
P value	0.00000+-0.0000	0.04505+-0.0203	0.39640+-0.0528	0.25225+-0.0424	0.27027+-0.0429	0.00000+-0.0000	0.00000+-0.0000	0.00000+-0.0000	0.00000+-0.0000	0.00000+-0.0000	0.06306+-0.0194	0.05405+-0.0201	0.32432+-0.0707	0.29730+-0.0273	0.15315+-0.0360
D18S51															
Fst	0.01752	0.01425	0.0156	0.00076	0.00202	0.02499	0.019	0.00264	0.00991	0.00785	0.01561	0.00225	0.00438	0.00161	0.04626
P value	0.00000+-0.0000	0.00000+-0.0000	0.00000+-0.0000	0.27928+-0.0628	0.05405+-0.0148	0.00000+-0.0000	0.00000+-0.0000	0.10811+-0.0264	0.00000+-0.0000	0.06306+-0.0139	0.00000+-0.0000	0.12613+-0.0364	0.00901+-0.0091	0.17117+-0.0212	0.00000+-0.0000
D5S818															
Fst	0.01321	0.0106	0.00805	0.00201	0.01076	-0.00084	0.00111	0.00148	0.00041	0.00179	0.02681	0.00895	0.0058	0.00281	0.01067
P value	0.00000+-0.0000	0.05405+-0.0201	0.09910+-0.0286	0.27928+-0.0417	0.00000+-0.0000	0.48649+-0.0388	0.30631+-0.0388	0.19820+-0.0297	0.34234+-0.0354	0.24324+-0.0273	0.00000+-0.0000	0.00000+-0.0000	0.00901+-0.0091	0.13514+-0.0279	0.00000+-0.0000
FGA															
Fst	0.00305	0.00079	0.00888	0.00364	0.00325	0.00242	0.09958	0.00813	0.21302	0.21675	0.00825	0.00541	0.01982	0.00135	0.02212
P value	0.06306+-0.0194	0.35135+-0.0438	0.02703+-0.0139	0.12613+-0.0388	0.03604+-0.0148	0.10811+-0.0264	0.00000+-0.0000	0.00000+-0.0000	0.00000+-0.0000	0.00000+-0.0000	0.02703+-0.0139	0.00901+-0.0091	0.00000+-0.0000	0.19820+-0.0353	0.00000+-0.0000
